# Distribution, Risk Factors and Epidemiological Trends of Pancreatic Cancer Across Countries’ Income Levels: A Comprehensive Analysis

**DOI:** 10.1002/cnr2.70154

**Published:** 2025-02-17

**Authors:** Sofia Laila Wik, Wenxin Tian, Claire Chenwen Zhong, Apurva Sawhney, Mingjun Gao, Qinyao Yu, Fanyu Xue, Sze Chai Chan, Shui Hang Chow, Yusuff Adebayo Adebisi, Jinqiu Yuan, Don Eliseo Lucero‐Prisno, Martin C. S. Wong, Junjie Huang, Global Health Focus Epidemiology Group (GHFEG)

**Affiliations:** ^1^ The Jockey Club School of Public Health and Primary Care, Faculty of Medicine, the Chinese University of Hong Kong Hong Kong SAR China; ^2^ Karolinska Institute Solna Sweden; ^3^ Centre for Health Education and Health Promotion, Faculty of Medicine, the Chinese University of Hong Kong Hong Kong SAR China; ^4^ Adam Smith Business School, College of Social Science, University of Glasgow Glasgow UK; ^5^ Jinan University‐University of Birmingham Joint Institute, Jinan University Guangzhou China; ^6^ School of Mathematics, College of Engineering and Physical Sciences, University of Birmingham Birmingham UK; ^7^ Faculty of Health Sciences, University of Ottawa Ottawa Canada; ^8^ Nuffield Department of Population Health University of Oxford Oxford UK; ^9^ Clinical Research Center, Big Data Center, the Seventh Affiliated Hospital, Sun Yat‐Sen University Shenzhen Guangdong China; ^10^ Department of Global Health and Development London School of Hygiene and Tropical Medicine London UK

**Keywords:** disease burden, low‐ and middle‐income countries, pancreatic cancer

## Abstract

**Background:**

Globally, pancreatic cancer poses a significant concern for public health.

**Aims:**

The objective of this study was to assess the burden of pancreatic cancer on varying income levels.

**Methods and Results:**

Data from the Global Burden of Disease Study (GBD) 2021 and Gross Domestic Product Per Capita data were utilised in this study. All countries were categorised into four groups based on their income levels. Age‐standardised incidence, mortality and disability‐adjusted life years (DALYs) rates were the primary parameters to analyse the burden of pancreatic cancer. The associations between pancreatic cancer burden and countries' economic levels were analysed with linear regression models. High‐income‐level countries generally had a higher burden compared to other income levels in 2021. Greenland had the highest rate of age‐standardised DALYs at 374.93 per 100 000, followed by Uruguay (297.06) and Monaco (290.87). A higher gross domestic product (GDP) per capita was linked to a higher age‐standardised incidence (β = 0.77, 95% CI = 0.63, 0.90, *p* < 0.001), mortality (β = 0.72, 95% CI = 0.59, 0.86, *p* < 0.001) and DALYs (β = 14.59, 95% CI = 11.38, 17.80, *p* < 0.001). From 1990 to 2021, the pancreatic cancer burden increased across all income levels, with the most pronounced rise seen in lower‐middle‐income countries. Smoking‐related age‐standardised DALYs have decreased since 1990. However, there was a notable increase in males in upper‐middle‐income countries during the same period.

**Conclusion:**

In conclusion, the pancreatic cancer burden has been increasing globally. The burden of pancreatic cancer varies significantly among countries with different income levels. Effective preventions are needed to control the burden of pancreatic cancer.

## 1 Introduction

Pancreatic cancer ranks among the most fatal cancers globally, exhibiting dismal 5‐year survival rates that range from 2% to 9% [1]. Pancreatic cancer was ranked eighth in mortality and 14th in incidence globally by the Global Burden of Diseases (GBD) 2016 [2]. However, this statistic varied considerably between income levels, with the highest rates of incidence and mortality being found in high‐income countries. Pancreatic cancer was ranked seventh in mortality and 12th in incidence from a study based on the GLOBOCAN database [3]. Approximately two‐thirds of the major risk factors linked to pancreatic cancer could be modified or altered [4]. Smoking is a strong risk factor for pancreatic cancer, accounting for estimated population‐attributable fractions of 11%–32% [4]. Furthermore, it is believed that the rising burden of pancreatic cancer can be attributed to the ageing population and lifestyle changes resulting from socioeconomic advancements [5].

The overall noncommunicable diseases (NCDs) burden and cancer burden showed different patterns when considering the socioeconomic level. In 2015, approximately 78% of global deaths attributable to NCDs, including cancers, took place in low‐ and middle‐income countries (LMICs). Of these, nearly 50% of deaths in LMICs were classified as premature death [6]. Cancer mortality continued to increase in LMICs compared to high‐income countries, where it was either declining or stable [7]. Cancer deaths in LMICs were projected to increase to 75% by 2030 [6]. These situations pose a major challenge for LMICs, as total and premature mortality will further lead to higher healthcare burdens, loss of productivity, and further economic impacts [5, 6].

Previous studies have explored pancreatic diseases using the same dataset in this study. Jiang et al. (2023) accessed the GBD 2019 for the age‐period‐cohort analysis of pancreatitis epidemiological trends [8]. Moreover, An et al. (2024) accessed the GBD 2019 database to obtain the incidence and the trend of pancreatic cancer [9]. Our study investigated different pathological conditions compared to Jiang et al. (2023) and added value in incorporating the gross domestic product (GDP) income levels from the World Bank dataset to provide a more comprehensive assessment of the burden of pancreatic cancer by comparison by the country's income level. Furthermore, our study also accessed the risk factor analysis, including smoking, elevated fasting plasma glucose and body mass index (BMI) in relation to the DALY rates, which were not included in the studies we mentioned above.

Therefore, this study aimed to use the GBD dataset to analyse the pancreatic cancer burden, with a focus on evaluating the pancreatic cancer burden by countries' income level, age group and sex. The primary objective is to investigate the burden and risk factors associated with pancreatic cancer to identify potential targets and suggestions for prevention strategies. By comprehensively assessing the burden of pancreatic cancer by GDP income levels, this research will deepen our understanding of the disease's impact, specifically in LMICs, and shed light on disparities in healthcare accessibility within these regions.

## 2 Materials and Methods

### 2.1 Data Source

The data regarding the burden of pancreatic cancer were sourced from the Global Health Data Exchange GBD Results Tool, accessible at https://vizhub.healthdata.org/gbd‐results/. The GBD collaborators developed this tool to facilitate a comprehensive analysis of age‐ and sex‐specific mortality across 288 causes, the prevalence and years lived with disability for 371 diseases and injuries, and the comparative risks associated with 88 risk factors. This evaluation spans 204 countries and territories, as well as 811 subnational locations, covering the period from 1990 to 2021 [10]. The protocol for GBD 2021 was published on the Institute for Health Metrics and Evaluation website [11]. All analyses conducted for GBD 2021 adhered to the Guidelines for Accurate and Transparent Health Estimates Reporting statement.

The World Bank Income levels were utilised to assess countries' economic statuses, with ‘GDP per capita (USD)’ data obtained from the World Bank dataset at https://data.worldbank.org/indicator. This allows for a standardised comparison of the impact of economic level on the burden of pancreatic cancer across countries. Income classification thresholds in 2021–2022 were as follows: low‐income countries earn less than $1045; lower‐middle‐income countries earn $1046–$4095; upper‐middle‐income countries earn $4096–$12 695; and high‐income countries earn $12 695 or more [12]. Some regions were excluded due to unavailable GDP data. The details on the classification of countries by per capita income are listed in Table S1.

### 2.2 Cancer Burden Indicators

Age‐standardised incidence, mortality and rate of disability‐adjusted life years (DALYs) were used to measure the burden of pancreatic cancer. DALYs take into account both premature mortality and the burden of disability caused by the disease, encompassing years of life lost (YLLs) and years lived with disability (YLDs). This metric quantifies the loss of healthy years equivalent to 1 year of full health, providing a comprehensive assessment of the burden ranging from conditions causing premature death with minimal disability to those resulting in significant disability without immediate fatality [13]. The correlation between age‐standardised incidence, mortality rates, DALYs rates and GDP was examined to understand the association between income levels and the burden of cancer. Temporal trends in pancreatic cancer burden were assessed using data spanning from 1990 to 2021, considering variations by World Bank Income levels and gender. For risk factor analysis, Level 4 risk factors such as tobacco usage, elevated fasting plasma glucose, and BMI were selected. The age‐standardised rate of DALYs was utilised to evaluate the temporal trends of pancreatic cancer burden attributed to each of these risk factors.

### 2.3 Statistical Analysis

This study focussed on the population aged 15 and above, as values for younger ages were negligible. The data used for analysing temporal trends dates back to 1990, enabling an in‐depth examination of patterns and evolving trends in pancreatic cancer burden across different income levels. This research further delved into the potential risk factors linked to pancreatic cancer. To comprehend their influence on the overall burden of this disease, the study evaluated the age‐standardised rates of DALYs associated with these risk factors. All data were available from the GHDx.

Besides, simple linear models were employed to calculate correlations between GDP and pancreatic cancer age‐standardised incidence, mortality and DALY rates. At the statistical level, a p‐value threshold of less than 0.05 was considered indicative of statistical significance. Statistical analysis and data visualisations were created using R software, enabling a clear and informative presentation of the observed trends and patterns.

## 3 Results

### 3.1 The Overall Burden of Pancreatic Cancer in 2021

There were disparities in age‐standardised DALYs rates for pancreatic cancer across different countries and regions, as shown in Figure S1. Greenland had the highest rate of age‐standardised DALYs at 374.93 per 100 000 in 2021, followed closely by Uruguay (297.06) and Monaco (290.87). On the other hand, countries such as Mozambique (19.30), Sao Tome and Principe (23.67) and Nigeria (25.25) exhibited the lowest age‐standardised DALYs rates for pancreatic cancer. High‐income‐level countries generally had a higher pancreatic cancer burden compared to other income‐level countries. In the low‐income countries, the age‐standardised rate of DALYs ranged from 34.58 in Ethiopia to 116.38 in Uganda. For lower‐middle‐income countries, the rate of DALYs ranged from 23.67 in Sao Tome and Principe to 204.85 in Cabo Verde. In the upper‐middle‐income countries, the rate of DALYs ranged from 38.85 in Namibia to 262.82 in Bulgaria.

### 3.2 Comparison of Pancreatic Cancer Burden by Country's Income Level

**FIGURE 1:**
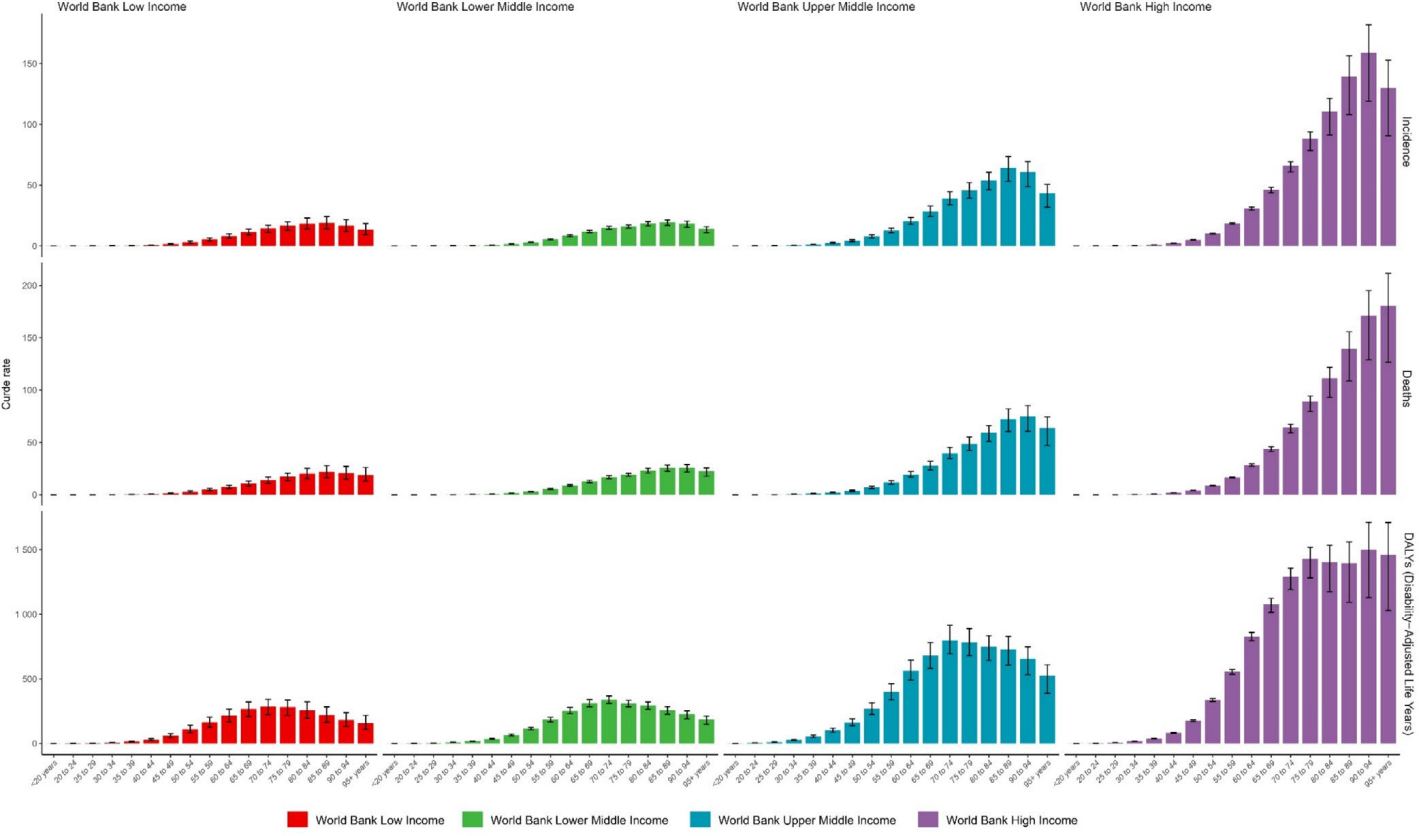
Age‐standardised incidence, mortality and DALYs for pancreatic cancer in 2021, by age group and income level.

Figure 1 illustrates the age‐standardised incidence, mortality and DALYs rate for pancreatic cancer across different age groups and income levels. In high‐income countries, rates of pancreatic cancer in each age group were generally higher compared to the other countries with lower‐income levels. The mortality burden was notably higher in high‐income countries, peaking at 95 years. Conversely, for other income levels, the highest mortality rates were observed in individuals older than 85 years. Regarding the rate of DALYs, the highest burden in the high‐income category was seen in the 91–94 age groups, at 1498.88 per 100 000. Across the remaining income brackets, the incidence of pancreatic cancer peaked at younger age groups: specifically, the 85–89 age group for upper‐middle‐income countries (64.31), lower‐middle‐income countries (19.54) and low‐income countries (18.91). Across all income groups, there was a trend towards higher burden in older age groups, with most peaks occurring beyond 85 years of age.

### 3.3 Relation Between Income Level and Pancreatic Cancer Incidence, Mortality and DALYs in 2021

**FIGURE 2:**
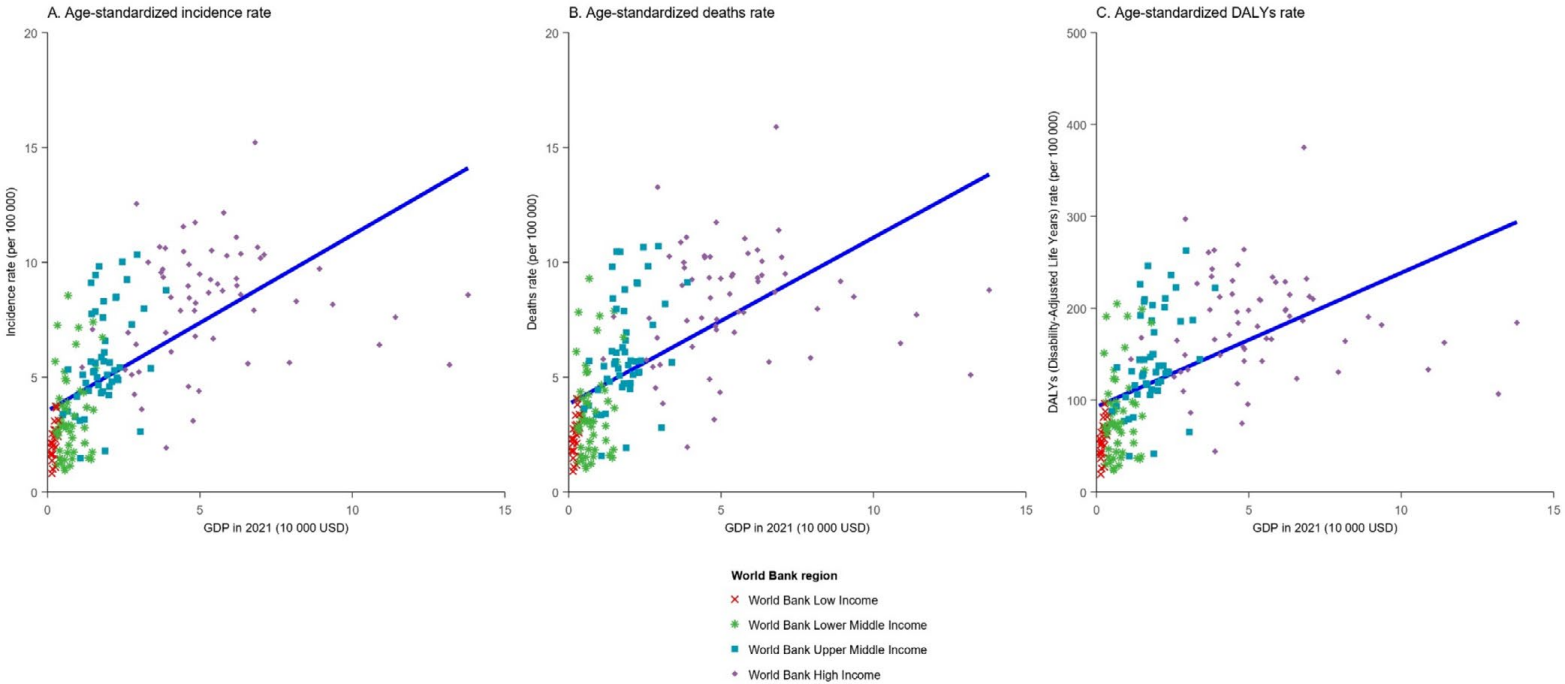
Scatterplot of GDP (in 10 000 USD) levels and age‐standardised incidence, mortality and DALYs rates of pancreatic cancer for 189 countries worldwide. (a) Incidence, (b) Mortality, (c) Disability‐adjusted life year. L, Low‐income countries; LM, low‐middle income countries; UM, upper‐middle income countries; H, high income countries; DALYs, disability‐adjusted life years.

Figure 2 displays the association between age‐standardised incidence, mortality and rate of DALYs for pancreatic cancer and GDP in 2021. A higher GDP per capita was linked to a higher age‐standardised incidence (β = 0.77, 95% CI = 0.63, 0.90, *p* < 0.001), higher mortality (β = 0.72, 95% CI = 0.59, 0.86, *p* < 0.001) and a higher rate of DALYs for pancreatic cancer (β = 14.59, 95% CI = 11.38, 17.80, *p* < 0.001; see Table S2). Upon stratification by the income level, the associations within each income level were examined. A positive association persisted, and the values of incidence, mortality and DALYs rate increased with higher GDP per capita. Significant associations were found in low‐income countries, upper‐middle‐income countries, and high‐income countries for age‐standardised incidence and mortality. Regarding age‐standardised DALYs, significant associations were only noted in low‐income countries and high‐income countries. Notably, all indicators showed more pronounced increases in low‐income countries as GDP per capita increased (refer to Table 1 and Figure S2).

**TABLE 1:** Linear regression for age‐standardised incidence, mortality and rate of DALYs and GDP in 2021, by income level (in 10 000 USD).

Indicator	Income level	Coefficient (*β*)	95% CI (*β*)	Intercept	*p*
Incidence	Low income	**4.12**	**0.14, 8.11**	**1.27**	**0.043**
	Lower‐middle income	0.95	−0.32, 2.21	2.63	0.138
	Upper‐middle income	**1.33**	**0.50, 2.15**	**3.37**	0.002*
	High income	0.12	−0.14, 0.38	7.57	0.356
Mortality	Low income	**4.32**	**0.08, 8.56**.	**1.40**	0.046*
	Lower‐middle income	0.88	−0.48, 2.23	2.91	0.200
	Upper‐middle income	**1.33**	**0.44, 2.21**	**3.73**	0.004*
	High income	0.08	−0.18, 0.34	7.82	0.525
DALYs	Low income	99.93	−2.12, 201.98	34.00	0.055
	Lower‐middle income	22.97	−9.78, 55.72	68.15	0.165
	Upper‐middle income	**32.95**	**13.15, 52.75**	**84.31**	0.002*
	High income	−0.03	−5.91, 5.84	182.35	0.991

*p‐value threshold of less than 0.05 was considered indicative of statistical significance.

### 3.4 Temporal Trend Analysis of Pancreatic Cancer Burden by Sex From 1990 to 2021

**FIGURE 3:**
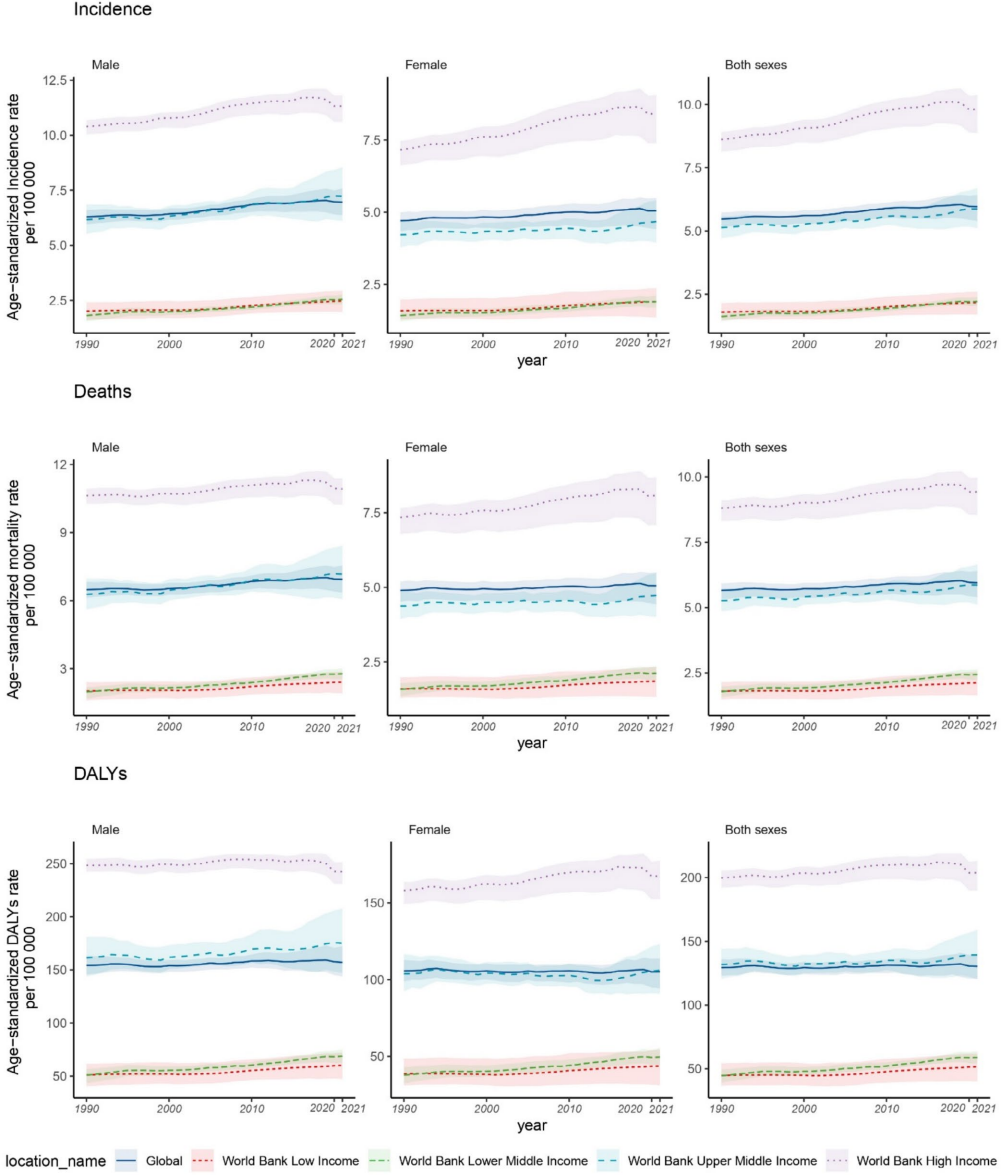
Temporal trends of age‐standardised incidence, mortality and DALYs rates by income level, 1990–2021. (a) males, (b) females, (c) Both sexes. DALYs, disability‐adjusted life years.

Figure 3 shows the temporal trends in pancreatic cancer burden from 1990 to 2021, revealing increasing trends in age‐standardised incidence, mortality and DALYs across all income levels during this period. There were regional differences in age‐standardised mortality and DALY rates for pancreatic cancer. The rates were highest in the World Bank high‐income group and lowest in the low‐income group for males, females and both sexes combined. A similar trend was observed in the age‐standardised incidence rates, although lower‐middle‐income countries showed rates comparable to low‐income countries. Regarding the temporal trend, the burden of pancreatic cancer has shown the most significant growth in lower‐middle‐income countries since 1990. Specifically, the incidence of pancreatic cancer has risen by 21% in low‐income countries, 37% in lower‐middle‐income countries, 14% in upper‐middle‐income countries and 14% in high‐income countries. Regarding mortality rates, lower‐middle‐income countries experienced a higher increment of approximately 37% since 1990, while lower‐income, upper‐middle‐income and high‐income countries saw increases of 18%, 11% and 7%, respectively. Additionally, the rates of DALYs have increased by 33% in lower‐middle‐income countries, 16% in low‐income countries, 6% in upper‐middle‐income countries and 2% in high‐income countries. Notably, the increments for females were relatively higher compared to males across all indicators for pancreatic cancer except in high‐income countries (Table S3).

### 3.5 Risk Factors for Pancreas Cancer

**FIGURE 4:**
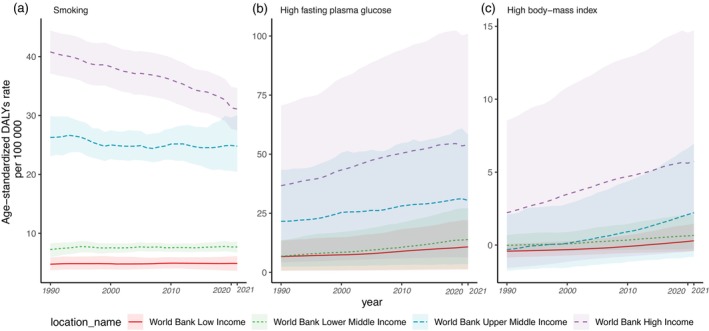
Age‐standardised DALYs rate of pancreatic cancer attributed to (a) smoking, (b) high fasting plasma glucose and (c) high body mass index for both sexes, from 1990 to 2021 by income group.

The risk factors for pancreatic cancer included in the study were smoking, elevated fasting plasma glucose and BMI. Figure 4 depicts the age‐standardised DALYs rates of pancreatic cancer for the entire population since 1990. In high‐income countries, the burden of pancreatic cancer related to smoking has decreased since 1990. However, this decreasing trend in smoking‐related burden was not addressed in the other countries based on income‐level categories. The reduction in smoking‐related age‐standardised DALYs rates was more noticeable for males in high‐income countries, while females remained relatively stable during the same period (Figure S3). For elevated fasting plasma glucose and BMI, there were no significant changes during the same time period for the total population, males and females, as observed in Figure S3. This indicated that the burden of pancreatic cancer associated with these two risk factors has remained relatively stable over the years across different demographic groups.

## 4 Discussion

This study assessed the burden of pancreatic cancer by the country's income level. In 2021, a concentration of pancreatic cancer burden was identified among elderly individuals in high‐income countries. Positive associations were found between age‐standardised incidences, mortality, and DALYs rates of pancreatic cancer with GDP, indicating a link between economic development and pancreatic cancer burden. However, since 1990, the highest increment in pancreatic cancer burden was found in lower‐middle‐income countries. Analysis of smoking‐related age‐standardised DALY rates revealed a decrease in the burden of pancreatic cancer related to smoking in high‐income countries since 1990, with a more prominent reduction observed in males compared to females. However, this decreasing trend was not observed in other income‐level countries. There were no significant changes in the burden of pancreatic cancer associated with elevated fasting plasma glucose and BMI over the same period for the total population, males and females across all income levels.

Overall, pancreatic cancer posed a greater burden in high‐income countries, with age‐standardised incidence, mortality, and DALY rates all decreasing as income levels decrease. As shown in the map, a notably darker red was shown in Central Europe, Eastern Europe and Russia indicating a high rate of DALYs, while Ethiopia, Somalia and Guinea exhibited a much lighter gradient of red, suggesting lower DALYs per capita. However, it is important to note that the lowest rate of DALYs in Central Africa may not fully reflect the actual situation and could be influenced by limitations in data collection that require further improvements. Other studies have found the same conclusion that the use of the Social Development Index (SDI) to reflect a country's socioeconomic level further strengthened the understanding of the association between socioeconomic status and the burden of pancreatic cancer, with areas with high SDI having the highest incidence and mortality rates, while areas with low and medium‐low SDI have lower morbidity and mortality rates [14]. These consistent patterns across studies provide robust evidence supporting the association between income levels, highlighting the need for targeted interventions and healthcare strategies tailoured to the specific socioeconomic contexts in which this disease manifests.

This study has uncovered that the heaviest burden of pancreatic cancer is concentrated primarily among the elderly population. This feature did not vary significantly when stratified by the income level. It is noteworthy that life expectancies followed a descending order, with high‐income countries exhibiting the highest life expectancy (81.0 years), followed by upper‐middle‐income (71.5 years), lower‐middle‐income (69.1 years) and low‐income countries (63.4 years). This observation indicated a relationship between the highest burden of pancreatic cancer and the life expectancy within a specific region [15]. In high‐income countries, the peak age for both incidence and mortality occurred approximately 10 years later compared to low‐income countries. The most prominent risk factor for pancreatic cancer is age, and as the life expectancy of the global population continues to grow, so does the overall prevalence of pancreatic cancer [16]. According to World Bank data, the proportion of elderly people aged over 65 years increased from 6.1% in 1990 to 9.2% in 2021, and it is projected to rise to 16% in 2050 [17, 18]. Given the dismal survival rate of pancreatic cancer, healthcare and early detection are of paramount importance, especially considering the anticipated demographic shift towards an older population.

The positive associations between GDP and the incidence, mortality and DALYs rates of pancreatic cancer on a country level in 2021 were evident. When stratified by the income level, statistically significant associations were found in both low‐income and high‐income countries, while low‐income countries showed the most significant increment with each $10 000 increase in GDP. Consistent with similar studies, pancreatic cancer emerged as predominantly affecting high‐income countries, where age‐standardised incidence, mortality and DALYs rates were notably higher compared to middle‐ and low‐income countries [5, 19, 20]. Since 1990, regions with high SDI exhibited the highest rates of pancreatic cancer, while regions with low SDI values had the lowest rates. There was a clear association between changing patterns of pancreatic cancer and countries' income levels, indicating a link between increasing trends of the incidence and mortality of pancreatic cancer and national development status as measured by SDI from 1990 to 2017 [20].

The findings regarding the increment in pancreatic cancer burden among lower‐middle‐income countries are indeed alarming and emphasise the urgent need for targeted interventions in these regions. The expected doubling of the population aged over 65 years in many lower‐middle‐income countries, coupled with the increasing trend of pancreatic cancer burden among younger individuals (15–49 years), underscores the complexity of the challenge [21]. Even without significant improvements in healthcare systems, it is anticipated that the incidence of pancreatic cancer in low‐ and lower‐middle‐income countries will continue to rise over the coming decades [16]. This worrisome trend is not confined to specific regions, as evidenced by global forecasts indicating a substantial increase in pancreatic cancer mortality worldwide [22]. Globally, pancreatic cancer mortality will increase from 405.50 per 1000 population in 2016 to 750.64 per 1000 in 2040 [23]. A study has projected that pancreatic cancer is anticipated to outpace breast, prostate and colorectal cancers, making it the second most significant cause of cancer‐related mortality in the United States by the year 2030 [24]. Another study forecasted the incidence of pancreatic cancer will increase from 12.1 per 100 000 in 2010 to 15.1 and 18.6 per 100 000 in 2030 and 2050 in New Zealand [25].

Females generally exhibited larger increases in pancreatic cancer rates than males across different income groups, except for the upper‐middle‐income group. These trends, which were also identified among younger women in America and potentially in other countries, highlight the particular vulnerability of women to pancreatic [26]. Despite males generally having higher rates of pancreatic cancer across all income groups during the analysis period, the significant increase among females suggests a shifting trend that warrants attention. Potential hormonal factors, such as the use of oral contraceptives (OCs), hormone replacement therapy (HRT) and age at menarche, have been implicated in contributing to the elevated risk of pancreatic cancer among females [27]. Several studies, encompassing a prospective cohort study in Japan, have documented a favourable correlation between the utilisation of exogenous hormones and an elevated risk of pancreatic cancer among women, whereas other reproductive factors among females did not exhibit a comparable association [28]. Understanding the underlying factors driving these gender disparities, whether related to risk factor prevalence, biological differences or healthcare‐seeking behaviours, is crucial for developing targeted interventions that can effectively address the increasing burden of pancreatic cancer among both males and females. By identifying and addressing these specific risk factors and considerations unique to each gender, healthcare systems and policymakers can implement more tailoured and impactful strategies to combat pancreatic cancer and improve outcomes for all individuals affected by this disease.

Smoking‐related age‐standardised DALYs show decreasing trends in high‐income countries; this decreasing trend was more obvious in males than in females. However, in other income levels, especially for upper‐middle‐income countries, the smoking‐related DALYs showed an increasing trend. The persistent rise in pancreatic cancer mortality rates has been attributed to the sustained increase in tobacco consumption over the extended period, with cumulative tobacco exposure predicting mortality rates for both sexes, as evidenced in Australia [29]. Notably, the implementation of smoking reduction initiatives in Australia has led to a reduction in the mortality rate from pancreatic cancer among males, emphasising the importance of tobacco control measures in mitigating the disease burden, which can be extended to females as well [29]. A pooled population‐based cohort study revealed distinct patterns in the association between smoking and pancreatic cancer risk, with increased risk observed in both sexes for current smoking compared to never smoking [30]. Former smoking and small cumulative doses were associated with increased risk only among females, suggesting the need for comprehensive risk factor assessments to understand the rising burden in females [30]. Although females showed a larger increment in pancreatic cancer burden since 1990, the risk‐related cancer burden did not show that much significant difference. Other risk factors need to be considered to understand the increasing burden on females. A study estimated the metabolic‐related pancreatic cancer burden by 2030. In 2021, North America and Central Europe, regions with high incomes, exhibited the highest age‐standardised mortality rates for metabolic‐related pancreatic cancer. Furthermore, it is anticipated that the burden associated with high FPG and high BMI will persistently increase over the next decade [21]. Since this study only included three risk factors without considering hormonal‐related factors, further analysis of the associations between familial predisposition and hormonal factors in females is necessary and effective target prevention strategies are needed [31]. On the other hand, we acknowledged the importance of assessing the effects of COVID‐19 on the burden of pancreatic cancer across different income levels. However, in GBD 2021, our ability to capture the specific indirect effects of COVID‐19 was limited, particularly regarding both fatal and nonfatal outcomes.

The current allocation for health services and policy research in LMICs averages roughly 0.007% of their overall healthcare expenditures, which is significantly lower than the recommended allocation of 0.1% [6, 32]. This discrepancy highlights the substantial gap between the recommended and actual investment in research for LMICs. Furthermore, the majority of LMICs do not have sufficient cancer registries available. According to the International Agency for Research on Cancer's global cancer incidence report, only a small percentage of regions have sufficient population‐based data for inclusion. Specifically, Africa has 1%, Asia has 4%, South and Central America have 4%, while North America boasts an inclusion rate of 80% [6, 33]. This disparity underscores the need for improved data collection and research infrastructure in LMICs to enhance our understanding of cancer incidence and facilitate evidence‐based policies and interventions. Challenges associated with treating pancreatic cancer arise from various factors, such as the pancreas's remote location, the absence of suitable screening tests or diagnostic markers, the aggressive nature of pancreatic adenocarcinoma, its limited response to chemotherapy or radiotherapy and the complexities involved in establishing a tissue diagnosis. These combined difficulties contribute to the low success rates observed in the treatment of pancreatic cancer [5, 34].

## 5 Conclusion

This study underscores the significant toll of pancreatic cancer across nations of varying income levels, revealing a heavier burden in high‐income countries and among older demographic groups. Females generally exhibited larger increases than males across all income groups, except for the upper‐middle‐income group. A positive association between countries' GDP levels and pancreatic cancer incidence, mortality and rate of DALYs was observed, and these associations were more significant in the high‐ and low‐income countries. Since 1990, the lower‐middle‐income group has exhibited distinct patterns that experienced the most substantial increase. Smoking‐related age‐standardised DALYs show decreasing trends in high‐income countries, while increasing in other income levels, especially for upper‐middle‐income countries. Socioeconomic factors and healthcare disparities are likely significant contributors to the vulnerabilities observed by the country's income level.

## Author Contributions

**Sofia Laila Wik:** data curation (lead), formal analysis (lead), writing – original draft (lead). **Wenxin Tian:** data curation (lead), formal analysis (lead), writing – original draft (lead). **Claire Chenwen Zhong:** supervision (lead), writing – review and editing (lead). **Apurva Sawhney:** writing – original draft (equal). **Mingjun Gao:** writing – original draft (equal). **Qinyao Yu:** writing – original draft (equal). **Fanyu Xue:** writing – original draft (equal). **Sze Chai Chan:** writing – original draft (equal). **Shui Hang Chow:** writing – original draft (equal). **Yusuff Adebayo Adebisi:** writing – review and editing (equal). **Jinqiu Yuan:** writing – review and editing (equal). **Don Eliseo Lucero‐Prisno III:** writing – review and editing (equal). **Martin C. S. Wong:** conceptualization (lead), supervision (lead), writing – review and editing (lead). **Junjie Huang:** conceptualization (lead), supervision (lead), writing – review and editing (lead).

## Ethics Statement

This study was approved by the Survey and Behavioral Research. Ethics Committee, The Chinese University of Hong Kong (No. SBRE‐22‐0826).

## Conflicts of Interest

The authors declare no conflicts of interest.

## Supporting information


Data S1.

## Data Availability

Publicly available datasets were analyzed in this study. All the data can be found from: GBD Results Tools (https://vizhub.healthdata.org/gbd‐results/).
