# Supplementary material for: Distribution, Risk Factors and Epidemiological Trends of Pancreatic Cancer Across Countries’ Income Levels: A Comprehensive Analysis
Source: Cancer Rep (Hoboken). 2025 Feb 17;8(2):e70154. doi: 10.1002/cnr2.70154 (PMC11830997; doi:10.1002/cnr2.70154)
Supplement: Supplementary file 1 — Data S1. [file CNR2-8-e70154-s001.docx]

**Supplementary Legends**

| **Supplementary Table 1.** | Classification of the per capita income of different groups of countries |
| --- | --- |
| **Supplementary Table 2.** | Linear regression for age-standardized incidence, mortality and DALYs rate and GDP (in 10,000 USD) in 2021 |
| **Supplementary Table 3.** | The percentage change for incidence, mortality and DALYs rate from 1990 to 2021. |
| **Supplementary Table 4.** | R codes for data analysis |
| **Supplementary Figure 1.** | World map of age-standardized DALYs rates for pancreatic cancer in 2021, both sexes |
| **Supplementary Figure 2.** | Scatterplot of GDP levels and age-standardized incidence, mortality and DALYs rates of pancreatic cancer for 189 countries, by income level region |
| **Supplementary Figure 3.** | Age-standardized DALYs rate of pancreatic cancer attributed to smoking, high fasting glucose and high body-mass index from 1990 to 2021 by income group |

**Supplementary Table 1.** Classification of the per capita income of different groups of countries in 2021

| Location name | Country Code | GDP per capita in 2021 | World Bank Income Level |
| --- | --- | --- | --- |
| Afghanistan | AFG | $2,138.87 | Low income |
| Albania | ALB | $16,261.80 | Upper Middle Income |
| Algeria | DZA | $14,690.92 | Lower Middle Income |
| Andorra | AND | $58,829.85 | High Income |
| Angola | AGO | $7,414.28 | Lower Middle Income |
| Antigua and Barbuda | ATG | $25,493.13 | High Income |
| Argentina | ARG | $26,086.80 | Upper Middle Income |
| Armenia | ARM | $16,899.07 | Upper Middle Income |
| Australia | AUS | $57,406.18 | High Income |
| Austria | AUT | $63,421.20 | High Income |
| Azerbaijan | AZE | $20,111.38 | Upper Middle Income |
| Bahamas | BHS | $28,510.91 | High Income |
| Bahrain | BHR | $54,351.84 | High Income |
| Bangladesh | BGD | $7,366.49 | Lower Middle Income |
| Barbados | BRB | $14,752.37 | High Income |
| Belarus | BLR | $27,611.18 | Upper Middle Income |
| Belgium | BEL | $62,123.16 | High Income |
| Belize | BLZ | $11,559.89 | Upper Middle Income |
| Benin | BEN | $3,575.31 | Lower Middle Income |
| Bermuda | BMU | $93,555.52 | High Income |
| Bhutan | BTN | $13,423.43 | Lower Middle Income |
| Bolivia (Plurinational State of) | BOL | $9,342.97 | Lower Middle Income |
| Bosnia and Herzegovina | BIH | $18,396.47 | Upper Middle Income |
| Botswana | BWA | $16,663.87 | Upper Middle Income |
| Brazil | BRA | $17,672.92 | Upper Middle Income |
| Brunei Darussalam | BRN | $79,364.09 | High Income |
| Bulgaria | BGR | $29,404.96 | Upper Middle Income |
| Burkina Faso | BFA | $2,467.74 | Low income |
| Burundi | BDI | $864.28 | Low income |
| Cabo Verde | CPV | $6,753.69 | Lower Middle Income |
| Cambodia | KHM | $4,668.24 | Lower Middle Income |
| Cameroon | CMR | $4,741.76 | Lower Middle Income |
| Canada | CAN | $55,781.70 | High Income |
| Central African Republic | CAF | $1,057.19 | Low income |
| Chad | TCD | $1,764.35 | Low income |
| Chile | CHL | $29,050.08 | High Income |
| China | CHN | $20,406.73 | Upper Middle Income |
| Colombia | COL | $17,587.23 | Upper Middle Income |
| Comoros | COM | $3,426.76 | Lower Middle Income |
| Congo | COG | $6,323.72 | Lower Middle Income |
| Costa Rica | CRI | $23,700.30 | Upper Middle Income |
| Côte d'Ivoire | CIV | $6,520.73 | Lower Middle Income |
| Croatia | HRV | $37,232.60 | High Income |
| Cyprus | CYP | $48,466.84 | High Income |
| Czechia | CZE | $48,418.12 | High Income |
| Democratic Republic of the Congo | COD | $1,358.20 | Low income |
| Denmark | DNK | $69,878.93 | High Income |
| Djibouti | DJI | $6,032.28 | Lower Middle Income |
| Dominica | DMA | $14,472.29 | Upper Middle Income |
| Dominican Republic | DOM | $21,923.30 | Upper Middle Income |
| Ecuador | ECU | $13,452.01 | Upper Middle Income |
| Egypt | EGY | $15,820.67 | Lower Middle Income |
| El Salvador | SLV | $10,709.76 | Lower Middle Income |
| Equatorial Guinea | GNQ | $18,100.91 | Upper Middle Income |
| Estonia | EST | $44,652.00 | High Income |
| Eswatini | SWZ | $10,202.51 | Lower Middle Income |
| Ethiopia | ETH | $2,628.34 | Low income |
| Fiji | FJI | $10,593.91 | Upper Middle Income |
| Finland | FIN | $57,788.90 | High Income |
| France | FRA | $53,834.80 | High Income |
| Gabon | GAB | $19,552.36 | Upper Middle Income |
| Gambia | GMB | $2,710.55 | Low income |
| Georgia | GEO | $18,915.65 | Upper Middle Income |
| Germany | DEU | $61,939.65 | High Income |
| Ghana | GHA | $6,544.24 | Lower Middle Income |
| Greece | GRC | $33,018.25 | High Income |
| Greenland | GRL | $68,086.46 | High Income |
| Grenada | GRD | $14,325.55 | Upper Middle Income |
| Guatemala | GTM | $12,106.42 | Upper Middle Income |
| Guinea | GIN | $3,735.09 | Low income |
| Guinea-Bissau | GNB | $2,278.02 | Low income |
| Guyana | GUY | $23,176.13 | Upper Middle Income |
| Haiti | HTI | $3,114.96 | Lower Middle Income |
| Honduras | HND | $6,209.94 | Lower Middle Income |
| Hungary | HUN | $38,648.00 | High Income |
| Iceland | ISL | $61,966.42 | High Income |
| India | IND | $8,087.99 | Lower Middle Income |
| Indonesia | IDN | $12,897.10 | Lower Middle Income |
| Iran (Islamic Republic of) | IRN | $15,042.37 | Lower Middle Income |
| Iraq | IRQ | $12,596.53 | Upper Middle Income |
| Ireland | IRL | $114,120.54 | High Income |
| Israel | ISR | $46,126.64 | High Income |
| Italy | ITA | $49,901.61 | High Income |
| Jamaica | JAM | $9,610.99 | Upper Middle Income |
| Japan | JPN | $44,549.34 | High Income |
| Jordan | JOR | $9,115.02 | Upper Middle Income |
| Kazakhstan | KAZ | $33,893.90 | Upper Middle Income |
| Kenya | KEN | $5,360.36 | Lower Middle Income |
| Kiribati | KIR | $3,036.42 | Lower Middle Income |
| Kuwait | KWT | $49,669.00 | High Income |
| Kyrgyzstan | KGZ | $5,802.39 | Lower Middle Income |
| Lao People's Democratic Republic | LAO | $8,111.06 | Lower Middle Income |
| Latvia | LVA | $36,777.89 | High Income |
| Lebanon | LBN | $11,860.06 | Lower Middle Income |
| Lesotho | LSO | $2,516.98 | Lower Middle Income |
| Liberia | LBR | $1,558.74 | Low income |
| Libya | LBY | $18,171.52 | Upper Middle Income |
| Lithuania | LTU | $46,410.07 | High Income |
| Luxembourg | LUX | $137,947.34 | High Income |
| Madagascar | MDG | $1,642.30 | Low income |
| Malawi | MWI | $1,728.83 | Low income |
| Malaysia | MYS | $30,456.09 | Upper Middle Income |
| Maldives | MDV | $18,865.72 | Upper Middle Income |
| Mali | MLI | $2,399.46 | Low income |
| Malta | MLT | $53,492.20 | High Income |
| Marshall Islands | MHL | $6,577.49 | Upper Middle Income |
| Mauritania | MRT | $5,988.20 | Lower Middle Income |
| Mauritius | MUS | $22,737.57 | Upper Middle Income |
| Mexico | MEX | $21,131.92 | Upper Middle Income |
| Micronesia (Federated States of) | FSM | $3,870.93 | Lower Middle Income |
| Mongolia | MNG | $14,951.14 | Lower Middle Income |
| Montenegro | MNE | $24,509.94 | Upper Middle Income |
| Morocco | MAR | $8,581.33 | Lower Middle Income |
| Mozambique | MOZ | $1,440.46 | Low income |
| Myanmar | MMR | $5,138.89 | Lower Middle Income |
| Namibia | NAM | $10,785.08 | Upper Middle Income |
| Nauru | NRU | $11,340.04 | High Income |
| Nepal | NPL | $4,461.47 | Lower Middle Income |
| Netherlands | NLD | $67,693.33 | High Income |
| New Zealand | NZL | $48,194.04 | High Income |
| Nicaragua | NIC | $6,873.73 | Lower Middle Income |
| Niger | NER | $1,538.47 | Low income |
| Nigeria | NGA | $5,623.66 | Lower Middle Income |
| North Macedonia | MKD | $22,371.50 | Upper Middle Income |
| Norway | NOR | $89,209.14 | High Income |
| Oman | OMN | $38,930.99 | High Income |
| Pakistan | PAK | $5,554.59 | Lower Middle Income |
| Palau | PLW | $15,781.98 | Upper Middle Income |
| Palestine | PSE | $5,663.12 | Lower Middle Income |
| Panama | PAN | $30,895.83 | High Income |
| Papua New Guinea | PNG | $3,992.94 | Lower Middle Income |
| Paraguay | PRY | $15,361.08 | Upper Middle Income |
| Peru | PER | $15,028.63 | Upper Middle Income |
| Philippines | PHL | $8,797.17 | Lower Middle Income |
| Poland | POL | $40,462.69 | High Income |
| Portugal | PRT | $38,783.01 | High Income |
| Puerto Rico | PRI | $40,559.70 | High Income |
| Qatar | QAT | $108,865.14 | High Income |
| Republic of Korea | KOR | $48,571.43 | High Income |
| Republic of Moldova | MDA | $15,682.07 | Upper Middle Income |
| Romania | ROU | $37,969.15 | High Income |
| Russian Federation | RUS | $38,938.50 | Upper Middle Income |
| Rwanda | RWA | $2,709.46 | Low income |
| Saint Kitts and Nevis | KNA | $26,420.34 | High Income |
| Saint Lucia | LCA | $18,651.87 | Upper Middle Income |
| Saint Vincent and the Grenadines | VCT | $15,673.74 | Upper Middle Income |
| Samoa | WSM | $6,074.69 | Lower Middle Income |
| San Marino | SMR | $65,717.89 | High Income |
| Sao Tome and Principe | STP | $5,703.25 | Lower Middle Income |
| Saudi Arabia | SAU | $47,754.43 | High Income |
| Senegal | SEN | $4,258.79 | Lower Middle Income |
| Serbia | SRB | $22,574.23 | Upper Middle Income |
| Seychelles | SYC | $29,979.83 | High Income |
| Sierra Leone | SLE | $1,623.98 | Low income |
| Singapore | SGP | $131,864.09 | High Income |
| Slovakia | SVK | $37,790.79 | High Income |
| Slovenia | SVN | $46,502.10 | High Income |
| Solomon Islands | SLB | $2,714.19 | Lower Middle Income |
| Somalia | SOM | $1,461.97 | Low income |
| South Africa | ZAF | $14,172.54 | Upper Middle Income |
| Spain | ESP | $43,620.60 | High Income |
| Sri Lanka | LKA | $14,316.14 | Lower Middle Income |
| Sudan | SDN | $3,420.45 | Low income |
| Suriname | SUR | $18,447.34 | Upper Middle Income |
| Sweden | SWE | $63,375.04 | High Income |
| Switzerland | CHE | $81,603.21 | High Income |
| Syrian Arab Republic | SYR | $2,914.53 | Low income |
| Tajikistan | TJK | $4,074.56 | Lower Middle Income |
| Thailand | THA | $20,280.93 | Upper Middle Income |
| Timor-Leste | TLS | $6,970.00 | Lower Middle Income |
| Togo | TGO | $2,646.83 | Low income |
| Tonga | TON | $6,742.46 | Upper Middle Income |
| Trinidad and Tobago | TTO | $27,631.68 | High Income |
| Tunisia | TUN | $12,159.05 | Lower Middle Income |
| Türkiye | TUR | $31,637.53 | Upper Middle Income |
| Tuvalu | TUV | $5,053.75 | Upper Middle Income |
| Uganda | UGA | $2,688.27 | Low income |
| Ukraine | UKR | $18,040.35 | Lower Middle Income |
| United Arab Emirates | ARE | $68,912.55 | High Income |
| United Kingdom | GBR | $52,841.63 | High Income |
| United Republic of Tanzania | TZA | $3,451.45 | Lower Middle Income |
| United States of America | USA | $71,055.88 | High Income |
| United States Virgin Islands | VIR | $46,238.06 | High Income |
| Uruguay | URY | $29,187.43 | High Income |
| Uzbekistan | UZB | $8,162.20 | Lower Middle Income |
| Vanuatu | VUT | $3,006.58 | Lower Middle Income |
| Viet Nam | VNM | $12,230.26 | Lower Middle Income |
| Zambia | ZMB | $3,526.51 | Low income |
| Zimbabwe | ZWE | $3,276.27 | Lower Middle Income |

**Supplement Table 2**. Linear regression for age-standardized incidence, mortality and DALYs rate and GDP (in 10,000 USD) in 2021. DALYs, disability-adjusted life years.

| **Indicators** | **Coefficient (*β*)** | **95% *CI*** | **Intercept** | ***P* value** |
| --- | --- | --- | --- | --- |
| Incidence | 0.77 | 0.63, 0.90 | 3.53 | < 0.001 |
| Mortality | 0.72 | 0.59, 0.86 | 3.83 | < 0.001 |
| DALYs | 14.59 | 11.38, 17.80 | 92.56 | < 0.001 |

**Supplementary Table 3**. The percentage change for incidence, mortality and DALYs rate from 1990 to 2021.

| **Indicators** | **Sex** | **Income level** | **Percentage change from 1990 to 2021** | **Lower value** | **Upper level** |
| --- | --- | --- | --- | --- | --- |
| Incidence | Both | Low income | 21% | 50% | -3% |
|  |  | Lower-middle income | 37% | 62% | 18% |
|  |  | Upper-middle income | 14% | 35% | -4% |
|  |  | High income | 14% | 17% | 9% |
|  | Male | Low income | 23% | 51% | -1% |
|  |  | Lower-middle income | 41% | 71% | 20% |
|  |  | Upper-middle income | 17% | 45% | -8% |
|  |  | High income | 9% | 12% | 5% |
|  | Female | Low income | 20% | 58% | -11% |
|  |  | Lower-middle income | 34% | 66% | 12% |
|  |  | Upper-middle income | 11% | 33% | -9% |
|  |  | High income | 18% | 22% | 11% |
| Mortality | Both | Low income | 18% | 45% | -5% |
|  |  | Lower-middle income | 37% | 63% | 18% |
|  |  | Upper-middle income | 11% | 31% | -7% |
|  |  | High income | 7% | 10% | 3% |
|  | Male | Low income | 20% | 48% | -3% |
|  |  | Lower-middle income | 41% | 71% | 21% |
|  |  | Upper-middle income | 14% | 40% | -10% |
|  |  | High income | 3% | 5% | 0% |
|  | Female | Low income | 16% | 52% | -14% |
|  |  | Lower-middle income | 34% | 66% | 13% |
|  |  | Upper-middle income | 8% | 29% | -11% |
|  |  | High income | 10% | 14% | 4% |
| DALY | Both | Low income | 16% | 45% | -8% |
|  |  | Lower-middle income | 33% | 58% | 14% |
|  |  | Upper-middle income | 6% | 26% | -12% |
|  |  | High income | 2% | 4% | -1% |
|  | Male | Low income | 18% | 47% | -6% |
|  |  | Lower-middle income | 35% | 65% | 15% |
|  |  | Upper-middle income | 8% | 35% | -16% |
|  |  | High income | -2% | 0% | -5% |
|  | Female | Low income | 13% | 50% | -16% |
|  |  | Lower-middle income | 31% | 63% | 10% |
|  |  | Upper-middle income | 2% | 24% | -17% |
|  |  | High income | 6% | 9% | 2% |

DALYs, disability-adjusted life years.

**Supplementary Table 4. R codes for data analysis**

**Figure 1**

ggplot(data,aes(x=age_name,y=val,fill=label)) +

geom_col(position = "dodge",width = 0.8)+

geom_errorbar(aes(ymin=lower, ymax=upper),position = "dodge", width = 0.3)+

facet_grid((measure_name~label),scales = "free_y")

**Figure 2**

ggplot(data, aes(x = GDP2021_10000, y = val)) +

theme_bw() +

geom_smooth(method = "lm", se = FALSE, color = "blue") +

geom_point(aes(shape = WBRegion, fill = WBRegion, size = WBRegion, colour = WBRegion))

**Figure 3**

ggplot(data,aes(x=year,y=val,color=location_name)) +

geom_line(aes(linetype=location_name))+

geom_ribbon(aes(ymin=lower, ymax=upper,fill=location_name),alpha=0.1,color=NA)+

facet_wrap(vars(sex_name),scales = "free_y")

**Figure 3**

ggplot(data_Dalys_four,aes(x=year,y=val,color=location_name)) +

geom_line(aes(linetype=location_name))+

geom_ribbon(aes(ymin=lower, ymax=upper,fill=location_name),alpha=0.1,color=NA)+

facet_wrap(vars(rei_name),scales = "free_y")

**Figure 4**

ggplot(PC_risk_Dalys_four,aes(x=year,y=val,color=location_name)) +

geom_line(aes(linetype=location_name))+

geom_ribbon(aes(ymin=lower, ymax=upper,fill=location_name),alpha=0.1,color=NA)+

facet_wrap(vars(rei_name),scales = "free_y")+theme_classic()

**SFigure 1**

ggplot()+

geom_sf(aes(group=NAME,fill=val))

**SFigure 2**

ggplot(data, aes(x = GDP2021_10000, y = val), group = WBRegion, color = WBRegion) +

theme_bw() +

geom_smooth(method = "lm", se = FALSE, aes(group = WBRegion, color = WBRegion)) +

geom_point(aes(shape = WBRegion, fill = WBRegion, size = WBRegion, colour = WBRegion))

**SFigure 3**

ggplot(data,aes(x=year,y=val,color=location_name)) +

geom_line(aes(linetype=location_name))+

geom_ribbon(aes(ymin=lower, ymax=upper,fill=location_name),alpha=0.1,color=NA)+

facet_grid(rei_name~sex_name)

**Table 1**

lm(val ~ GDP2021_10000, data)


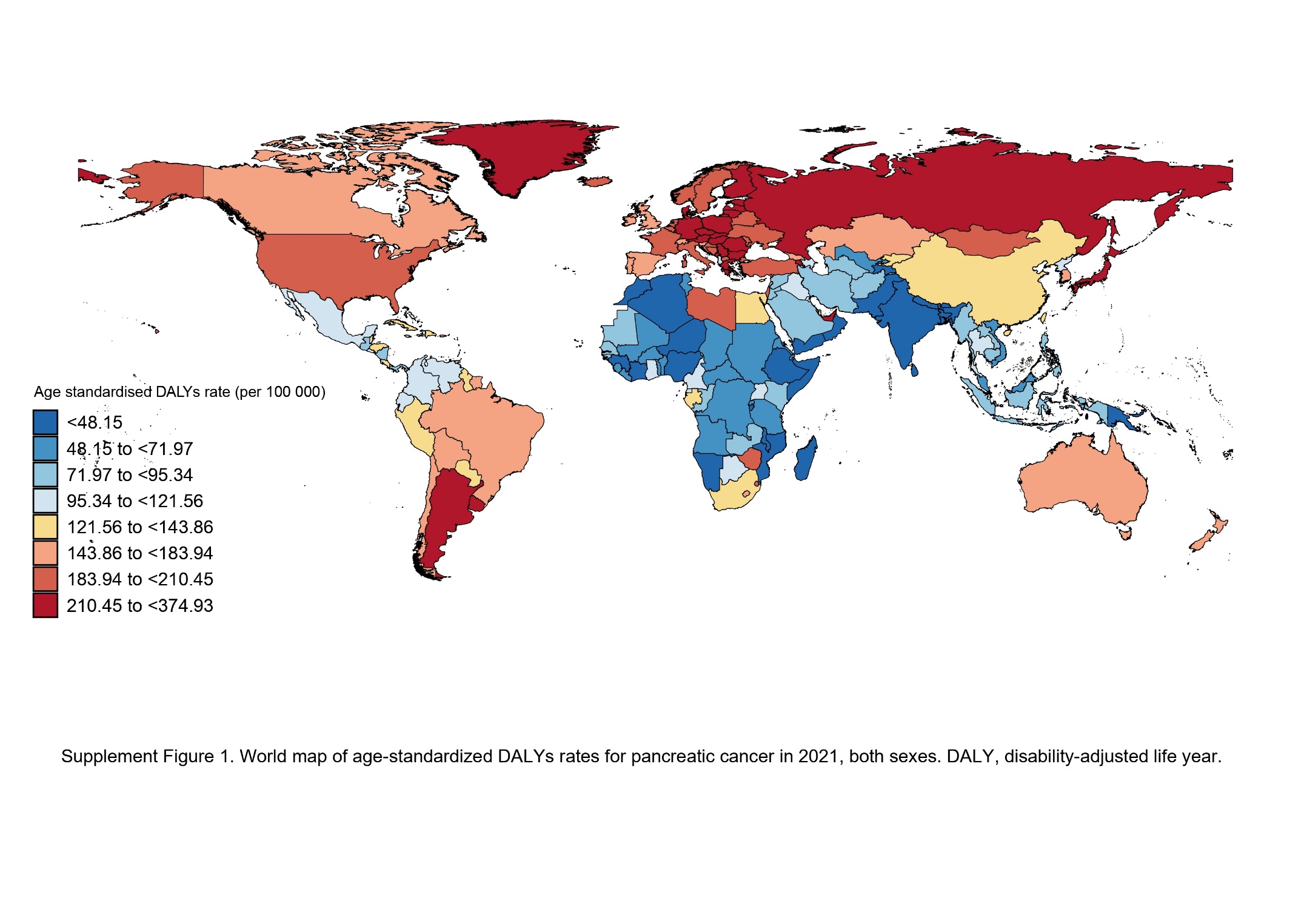


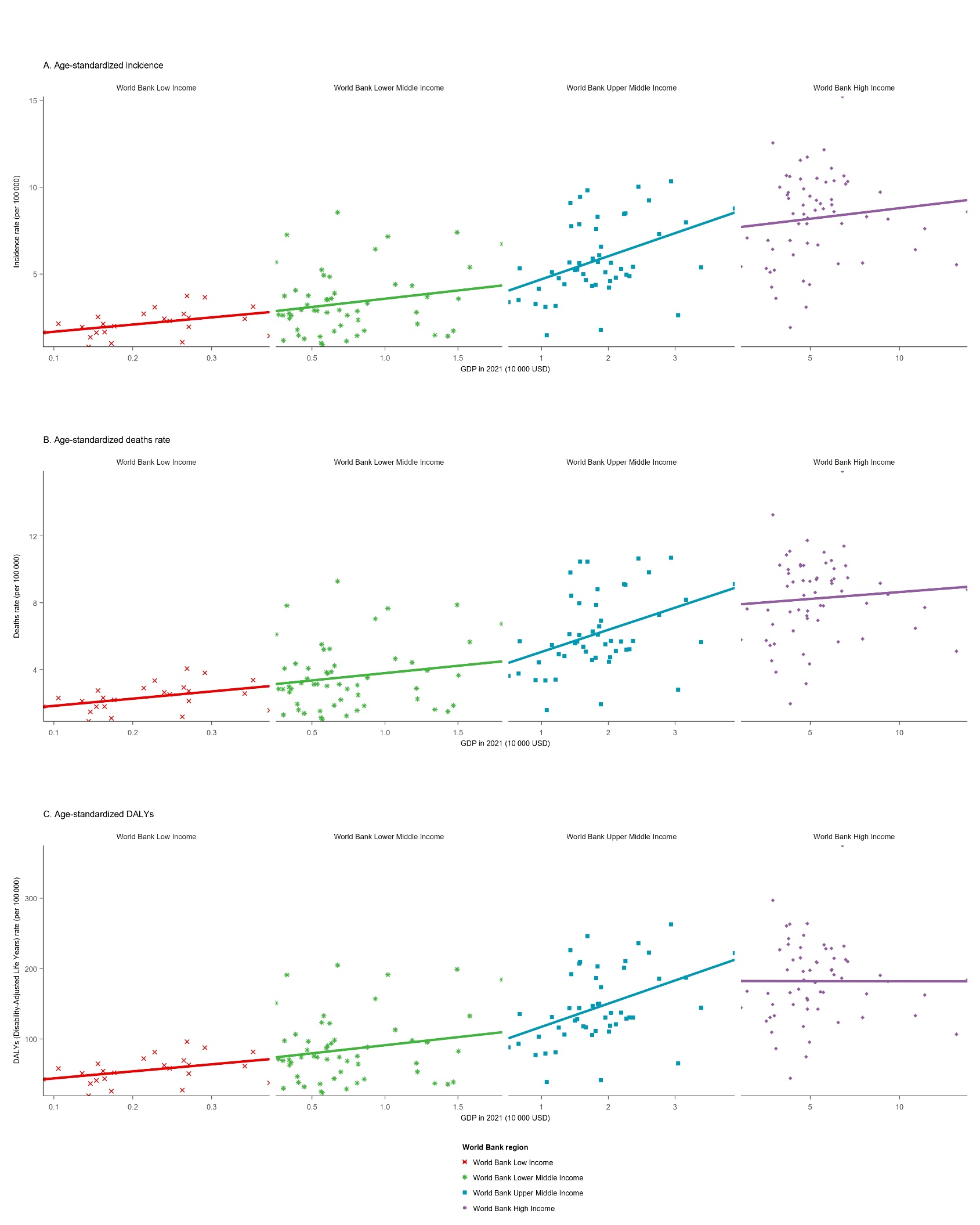


Supplementary Figure 2. Scatterplot of GDP levels and age-standardized incidence, mortality and DALYs rates of pancreatic cancer for 189 countries, by income level region.

GDP levels was downloaded from the World Bank website, 2021 version.

DALYs, disability-adjusted life years.


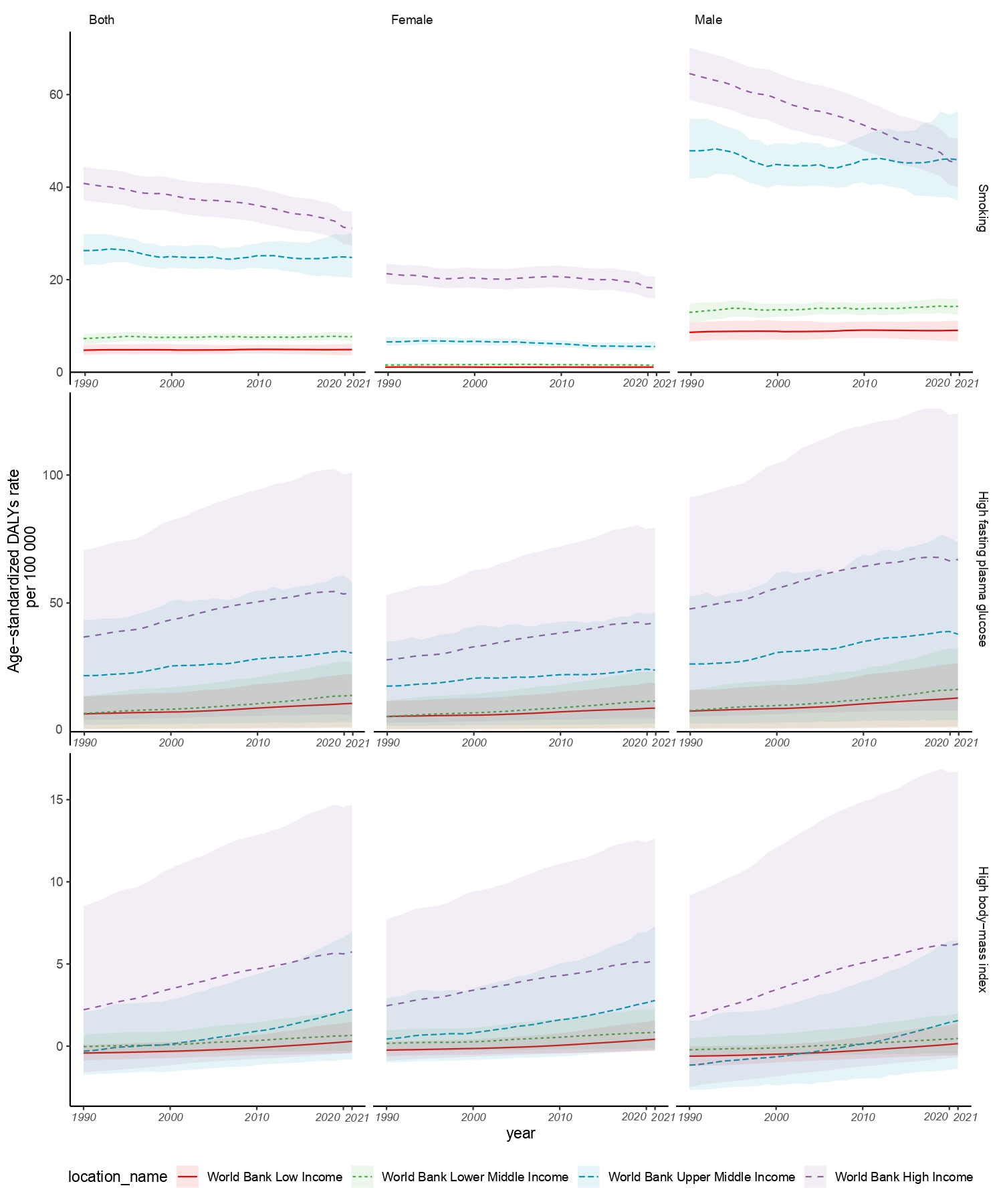


Supplementary Figure 3. Age-standardized DALYs rate of pancreatic cancer attributed to smoking, high fasting glucose and high body-mass index from 1990 to 2021 by income group. DALYs, disability-adjusted life years.
